# *Bacillus velezensis* Strain HN-Q-8 Induced Resistance to *Alternaria solani* and Stimulated Growth of Potato Plant

**DOI:** 10.3390/biology12060856

**Published:** 2023-06-14

**Authors:** Xuefei Bai, Qian Li, Dai Zhang, Yi Zhao, Dongmei Zhao, Yang Pan, Jinhui Wang, Zhihui Yang, Jiehua Zhu

**Affiliations:** College of Plant Protection, Hebei Agricultural University, Baoding 071000, China; baixuefei1996@126.com (X.B.); qianli_1009@126.com (Q.L.); adaiadai.1987@163.com (D.Z.); zhaoyi9323@163.com (Y.Z.); zhaodongm03@126.com (D.Z.); pyasd@sina.com (Y.P.); jinhwang@hebau.edu.cn (J.W.)

**Keywords:** potato, *Bacillus velezensis*, induced resistance, potato early blight, *Alternaria solani*, growth-promoting effect

## Abstract

**Simple Summary:**

The study found that *Bacillus velezensis* HN-Q-8 has an antagonistic effect on *Alternaria solani*. After being pretreated with strain HN-Q-8, which can induce potato plant resistance to early blight by regulating defensive enzyme activities and trig-gering the JA/ET pathway, as well as promote growth by regulating the contents of indole acetic acid, gibberellic acid 3 and abscisic acid, enhancing the chlorophyll content, and stimulating root activity. In addition, live bacteria and secondary metabolites should be used together for the best results. Thus, *B. velezensis* HN-Q-8 strain is an effective bacterial biocontrol agent, and our study can provide vital information for the research and development of biocontrol bacteria.

**Abstract:**

*Bacillus velezensis* HN-Q-8, isolated in our previous study, has an antagonistic effect on *Alternaria solani*. After being pretreated with a fermentation liquid with HN-Q-8 bacterial cell suspensions, the potato leaves inoculated with *A. solani* displayed smaller lesion areas and less yellowing than the controls. Interestingly, the activity levels of superoxide dismutase, peroxidase, and catalase in potato seedlings were enhanced by the addition of the fermentation liquid with bacterial cells. Additionally, the overexpression of key genes related to induced resistance in the Jasmonate/Ethylene pathway was activated by the addition of the fermentation liquid, suggesting that the HN-Q-8 strain induced resistance to potato early blight. In addition, our laboratory and field experiments showed that the HN-Q-8 strain can promote potato seedling growth and significantly increase tuber yield. The root activity and chlorophyll content of potato seedlings were significantly increased along with the levels of indole acetic acid, gibberellic acid 3, and abscisic acid upon addition of the HN-Q-8 strain. The fermentation liquid with bacterial cells was more efficient in inducing disease resistance and promoting growth than bacterial cell suspensions alone or the fermentation liquid without bacterial cells. Thus, the *B. velezensis* HN-Q-8 strain is an effective bacterial biocontrol agent, augmenting the options available for potato cultivation.

## 1. Introduction

Potato tubers (*Solanum tuberosum* L.) constitute one of the most widely consumed crops worldwide. They have the advantages of high availability, low price, and high nutritional value. As one of the most common and severe diseases in all potato-growing regions [1], potato early blight is caused by the necrotrophic pathogen *Alternaria solani*, resulting in leaf loss and reducing tuber yields by 30% in severe cases [2]. Chemical pesticides are still being used to prevent this [1]. However, the long-term use of such agents is environmentally harmful [3]. Therefore, an environmentally friendly control method is needed to help control potato early blight.

Studies have shown that the use of plant-growth-promoting rhizobacteria (PGPR) as biological control agents in agriculture is a successful plant disease control strategy [3]. It is well known that PGPR can inhibit the growth of pathogenic bacteria through the production of various secondary metabolites, such as cellulase [4], siderophores [5], volatile organic compounds [6], and lipopeptides [7,8,9,10]. In addition, PGPR can induce plant systemic resistance, which can reduce infections by pathogens [11,12]. Two defense mechanisms against pathogen infection have been confirmed in plants: induced systemic resistance (ISR) and systemic acquired resistance (SAR) [13,14]. Generally, PGPR often utilizes Jasmonate/Ethylene (JA/ET) signaling pathways to regulate plant ISR [15,16,17] and to protect plants against necrographic pathogens [18]. Furthermore, PGPR also stimulates the activity of plant defense enzymes, including superoxide dismutase (SOD), peroxidase (POD), and catalase (CAT) [19], thus participating in plant defense responses against pathogens. In addition, PGPRs can increase nutrient acquisition and regulate phytohormone levels [20], thereby promoting plant growth and improving crop yield [21,22,23]. 

As an important genus among PGPRs, *Bacillus* strains can produce a series of secondary metabolites in the growth process, thereby affecting the growth of plant pathogens [24] and activating plant ISR. For instance, the *Bacillus velezensis* SDTB038 can produce secondary metabolites (surfactin and fengycin) that help potato plants resist late blight development and can effectively inhibit the infection of potato leaves by *Phytophthora infestans* [25]. Similar effects have been observed for bacteria. Wang et al. [26] revealed that treatment with a suspension of *Bacillus subtilis* WXCDD105 in the rhizosphere of tomato enhanced the resistance to tomato gray mold and tomato leaf mold, and promoted the growth of tomato seedlings. In addition, both bacterial cell suspensions and fermentation liquid with and without bacterial cells inhibited the spore germination of wheat stripe rust and increased wheat resistance [27]. Although some secondary metabolites with disease resistance and growth-promoting effects have been identified [28], whether the main components of disease resistance and growth-promoting effects in the fermentation broth of *Bacillus* strains are the bacterial cells or secondary metabolites remains unclear. 

Previously, we screened a strain of *B. velezensis*, which has an antagonistic effect on *A. solani*, and named it HN-Q-8. Here, we further investigate the mechanism by which the *B. velezensis* HN-Q-8 strain induces resistance to the early blight of potato and promotes the growth of potato plants. The main research contents include: (1) The mechanism of induced resistance was investigated by detecting the changes of the antioxidant defense system and resistance-related genes; (2) The changes of plant hormones, root activity, and chlorophyll content were detected to explore the mechanism of promoting plant growth. Laboratory experiments showed that the different components of the *B. velezensis* HN-Q-8 strain could stimulate the defense enzyme activities of potato seedlings and trigger JA/ET pathways, which can induce the resistance of potato seedlings to early blight. In addition, a significant growth-promoting effect was observed with this strain, and the biomass of potato seedlings was increased by improving the root activity and chlorophyll content. Additionally, field test results showed that the *B. velezensis* HN-Q-8 strain not only promoted the growth of potted potato seedlings, but also increased the yield of potatoes. Overall, our work suggests that the *B. velezensis* HN-Q-8 strain can control early blight in potato plants, thus providing a novel biocontrol agent for potato production.

## 2. Materials and Methods

### 2.1. Potato Cultivars, Early Blight Strain, and B. velezensis Strain

Seed potatoes (cultivar Helan 15) with high susceptibility to *A. solani* were provided by Huigu Agricultural Company (Shijiazhuang City, Hebei Province, China). The pathogenic fungus *A. solani* strain HWC-168 was isolated and purified from diseased potato leaves [29]. The *B. velezensis* strain HN-Q-8 was screened, isolated, and purified from potato-rhizosphere-rich soil, as described below.

### 2.2. Preparation of B. velezensis and Pathogenic Fungi

The *B. velezensis* strain HN-Q-8 solution was strewed on a Luria–Bertani medium (LB medium) and placed in an incubator at 37 °C for 24 h. Single colonies of the HN-Q-8 strain were inoculated into Luria–Bertani liquid medium and cultured at 37 °C with a rotation of 200 rpm. When the cells reached 10^8^ colony-forming units (CFU mL^–1^), the culture was used as the fermentation liquid with bacterial cells (FLBS). After centrifuging the cultures (12,000 rpm at 4 °C for 20 min), the supernatant was harvested as the fermentation liquid without bacterial cells (FL), and the precipitation was resuspended with sterile water of the same volume as the bacterial solution as the bacterial cell suspension (BCS). All experiments used sterile water as a control (CK).

*A. solani* HWC-168 was inoculated on a tomato medium and cultured in a 25 °C incubator in the dark for 7 days. After mycelium filled the whole petri dish, *A. solani* HWC-16 hyphae were scraped off the medium with a sterilized blade and irradiated under UV light for 10 min. After the strain was incubated at 25 °C for 5 days with alternating 12 h light and 12 h dark periods, its spores were suspended in sterile water containing 0.1% Tween 80, and the spore concentration was adjusted to reach 10^5^ conidia·mL^−1^ as a pathogenic fungal preparation.

### 2.3. Soil Pretreatment and Potato Planting Methods

The experimental soil was the topsoil (upper 10 cm) of the experimental field at Hebei Agricultural University, Baoding City, Hebei Province, P. R. China. After removing impurities, using a high-pressure sterilization pot, it was sterilized 3 times, dried in a ventilated place, passed through a 100-mesh sieve, and stored at 4 °C for standby.

The seed potatoes were sterilized with 70% ethanol and rinsed. The sprouted seed potatoes were then cut into tubers with buds and transferred to the pot (20 cm diameter × 18 cm high) filled with soil for cultivation. The greenhouse temperature was maintained at 25 °C, the average daily sunlight time was 16 h, and the relative humidity was 40%.

### 2.4. Preinoculation with Strain HN-Q-8 and Inoculation of Pathogenic Fungus

Forty pots of one-month-old potato plants were used for biocontrol assays. Five milliliter aliquots of FLBS, FL, or BCS were applied carefully into the soil near the roots of the potato plant using a pipette, with ten repetitions per treatment. The same amount of sterilized water was used as the control (CK). All treatments were divided into two groups. Samples from one treatment group were taken at 0, 24, 48, and 72 h to measure the antioxidant enzyme activity and the relative expression of genes involved in the induced systemic resistance (ISR) signaling pathway, respectively. The other group was subjected to the *A. solani* challenge. Leaves were collected in the same position from the leaf crown of a potato plant for antifungal assays in vivo, and 20 μL 10^5^ conidia·mL^–1^ aliquots of *A. solani* were inoculated onto the potato leaves with the addition of FLBS, BCS, or FL. The necrotic areas of the leaves were measured 7 days after inoculation. The necrotic area = length of diseased spot × width of diseased spot × π/4.

### 2.5. Defensive Enzyme Activity Assays

At each sampling, three biological samples were collected from similar positions on the potato plant leaf crowns of the treatment and control groups. An amount of 0.1 g of the leaf was homogenized in 2 mL of 0.1 M sodium phosphate buffer (pH of 7.0) and centrifuged at 10,000× *g* for 25 min at 4 °C; the supernatant was used as an enzyme source. The activities of the SOD, POD, and CAT in the samples were determined by spectrophotometry, as described [30,31].

### 2.6. Growth-Promotion Test

Potato seedlings with four or five leaves were selected for growth-promoting-effects tests, and each treatment contained five plants. Five milliliter aliquots of FLBS, BCS, or FL were applied to the soil near the roots using a pipette. The same volume of sterile water was added to the control plants (CK). All treatments were divided into two groups, with five repetitions per group. Samples from one treatment group were taken at 0, 24, 48, and 72 h to examine the indole acetic acid (IAA), gibberellic acid3 (GA_3_), and abscisic acid (ABA) contents, and the relative expression levels of their related genes. The other group was used to measure the chlorophyll content, biomass, and root activity.

### 2.7. Determination of Potato Leaf IAA, GA_3_, and ABA Contents

The contents of the IAA, GA_3_, and ABA were determined by liquid chromatography/tandem mass spectrometry (LC/MS); the mass spectrometry parameters are shown in Table 1. Aliquots of 100 mg of potato plant leaves were ground under liquid nitrogen, and then dissolved in a 10 mL methanol–formic acid (99:1) solution, which were further subjected to ultrasonic oscillation for 2 min, and held at 4 °C for 12 h. The mixture was centrifuged at 10,000 rpm for 10 min. An amount of 1 milliliter of the supernatant was diluted to 10 mL with deionized water, and the diluent was loaded and adsorbed using a C18 solid-phase extraction column and washed with 6 mL of a 10% methanol solution. Finally, the product was eluted with 6 mL of the methanol–formic acid (99:1) solution and concentrated to 1 mL. Aliquots of 10 microliters of the resulting solution were analyzed in an LC/MS system (Shimadzu, Kyoto, Japan) using Agela Bonshell C18 (2.1 × 50 mm, 2.7 μm) columns.

### 2.8. Gene Expression Analysis of the ISR Signaling Pathway and Growth-Related Factors

Potato plant leaves collected for biocontrol assays and growth-promoting-effects tests were used to detect the gene expression levels in the ISR signaling and growth-related pathways, respectively. Total RNA from the potato plant leaves was extracted using the TRNzol Universal reagent (TianGen Biotech, Beijing, China). The cDNA was synthesized from RNA using a TransScript All-in-One First-Strand cDNA Synthesis SuperMix (TransGen Biotech, Beijing, China) for quantitative reverse-transcription polymerase chain reaction (RT-qPCR) amplification. We measured the transcription levels of the JA biosynthesis-related gene (Lipoxygenase, *StLOX2.1*), the ET biosynthesis-related gene (1-aminocyclopropane-1-carboxylate oxidase, *StACO1*), the resistance regulatory gene (nonexpressor of pathogenesis-related genes 1, *StNPR1*), the auxin response factor (Auxin response factors, *StARF2*), GA_3_ biosynthesis-related gene (Gibberellin 20-oxidases, *StGA20ox1*), and the ABA biosynthesis-related gene (9-cis-epoxycarotenoid dioxygenase, *StNCED1*) using a Bio-Rad Laboratories LightCycler^®^ 96 (Hercules, CA, USA). The *StActin7* gene was used as the internal control. The primer sequences for the RT-qPCR are listed in Table 2. All assays were performed synchronously in triplicate under the 2 × SuperFast Universal SYBR Master Mix method (ComWin Biotech Co., Ltd., Beijing, China). The RT-qPCR gene expression data were analyzed using the 2^−ΔΔCT^ method according to a previous study [32].

### 2.9. Determination of Chlorophyll Content, Biomass, and Root Activity

After the FLBS, FL, or BCS strains had been added to potato seedlings for 0, 7, 15, 22, and 30 days, the chlorophyll contents of the leaves at the same positions on the plants were measured using a chlorophyll soil and plant analyzer development tool (SPAD-502 PLUS, Konica Minolta, Tokyo, Japan).

After being treated with FLBS, BCS, or FL for 30 days, the root length (RL), lateral root number (LR), stem diameter (SD), plant height (H), dry weight (DW), and fresh weight (FW) of the potato seedlings were measured. Root activity was reflected by plant dehydrogenase (PDHA) activity, detected by Tetrazolium staining [33].

### 2.10. Effect of Strain HN-Q-8 on Potato Yield

Field experiments were performed at the Potato Resistance Identification Center of Baoding City, Hebei Province, to investigate the effects of the fermentation liquid with bacterial cells and its different components on the potato yield. All treatments were performed in a completely randomized block design with three replicates per treatment. Each plot area was about 100 m^2^, and the potato spacing was 20 cm. After the potato seedlings emerged, the FLBS, BCS, or FL samples were diluted to 360 mL per plot and irrigated. Water alone was added to the control plants. The potato tubers were harvested after 3 months of growth and weighed. A five-point sampling method was adopted during weighing, and twenty potato plants were collected from each plot. The calculations of the yield changes were as follows:

1. Total increase rate of tuber yield = ((total yield in the treatment area − total yield in the control area)/total yield in the control area) × 100%;
2. Increase rate of commercial potato tuber yield = ((commercial potato tuber yield in the treatment area − commercial potato tuber yield in the control area)/commercial potato tuber yield output in the control area) × 100% (potato tubers of >200 g were considered as of commercial value).

### 2.11. Statistical Analysis

Statistical analyses were performed using IBM SPSS Statistics 18 (IBM Corp., Armonk, NY, USA). All data were subjected to an analysis of variance (ANOVA). The mean values were compared using the Tukey-family error test at *p* < 0.05.

## 3. Results

### 3.1. Strain HN-Q-8 Induced the Resistance of Potato Leaves to Early Blight

**Figure 1:**
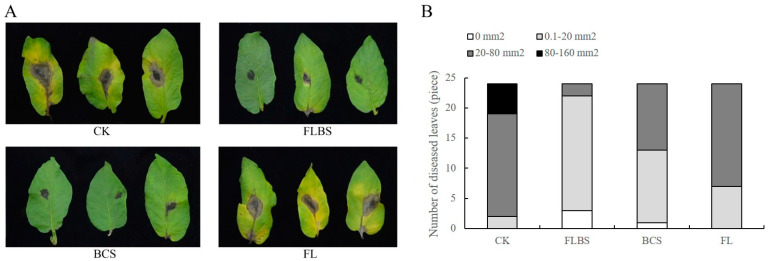
Effects of the fermentation liquid with strain HN-Q-8 bacterial cells (FLBS), bacterial cell suspension (BCS), or FL alone on early blight in isolated leaves inoculated with *A. solani.* (**A**) Images of the control effect of FLBS, BCS, or FL on early blight. (**B**) The distribution of necrotic areas around infected sites of plants inoculated with *A. solani* in different treatment groups and the control group.

To test the effect of the HN-Q-8 strain on the resistance to early blight in potato, using the method of leaf inoculation, the *A. solani* spore suspension was inoculated onto potato leaves pretreated with the fermentation liquid with bacterial cells (FLBS), the fermentation liquid without bacterial cell suspension (BCS), or the fermentation liquid without bacterial cells (FL). After 7 days of inoculation, we observed the lesioned areas of leaves with different treatments and the color changes around the lesions and found that the *B. velezensis* HN-Q-8 can induce resistance to *Alternaria solani*. As shown in Figure 1A, a large area of necrosis was formed on the untreated potato leaves inoculated with *A. solani*: the maximum necrotic area was 153 mm^2^. Obvious yellowing also appeared around the lesions (Figure 1B). Interestingly, treatments with FLBS and BCS caused reduced areas of lesions in the leaves following the inoculation of *A. solani* (Figure 1A), and all the areas were <80 mm^2^, among which 0.1–20 mm^2^ accounted for 92% and 54% of the total lesions, respectively. However, the leaves treated with FL inoculated with *A. solani* displayed larger lesions than those treated with FLBS or BCS, with most lesions measuring 20–80 mm^2^ and with more pronounced yellowing (Figure 1A). Thus, FLBS and BCS could induce a strong resistance to early blight in potato plants, but treatment with FL alone was ineffective.

### 3.2. Strain HN-Q-8 Stimulates Potato Defensive Enzyme Activities

**Figure 2:**
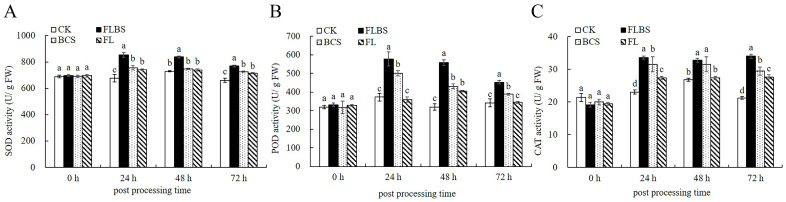
Effects of FLBS, BCS, or FL on the activities of superoxide dismutase (SOD) (**A**), peroxidase (POD) (**B**), and catalase (CAT) (**C**) in potato plants. Bars represent the standard error of three independent experiments. The results are presented as the means ± SDs (*n* = 3); columns labeled with different lowercase letters indicate statistically significant differences among treatments (*p* < 0.05).

Defensive enzymes in plants, such as superoxide dismutase (SOD), peroxidase (POD), and catalase (CAT), have been confirmed to be involved in defense responses against plant pathogens [34]. Here, the activities of these enzymes in potato plant leaves were measured after being pretreated with FLBS, BCS, or FL. As shown in Figure 2A–C, after the FLBS treatment, SOD and POD were significantly induced after 24 h, and reached peak activities at 24 h, with increases of 25.99% and 74.78%, respectively, with a slight decrease afterwards. However, compared with the control, there was significant induction up to 72 h. After 24 h of induction, CAT activity remained stable, fluctuated slightly, and the activity level increased by 60.13%. After the plants were treated with BCS, the activities of SOD, POD, and CAT in leaves showed a significant induction trend of increasing and then decreasing, and the activity was strongest at 24 h. The activities of the SOD, POD, and CAT were increased by 11.89%, 34.78%, and 38.56%, respectively. After treating the potatoes with FL, the changes of the SOD, POD, and CAT activities in potato leaves were small. Compared with the control, the SOD activity was the highest at 24 h, with an increase of 9.5%, and the POD activity was only significantly different from the control at 48 h, with an increase of 26.96%. The CAT activity had the greatest difference from the control at 72 h, when it was enhanced by 30.07%. Thus, treatments with FLBS, BCS, or FL could improve the activities of these three defense enzymes in potato plants. The effect of FLBS to induce the defense enzyme activity was the greatest, followed by BCS, and the effect of FL was weak.

### 3.3. ISR-Related Gene Expression Analysis

Beneficial microorganisms can enhance plant resistance to necrotrophic pathogens by triggering the plant JA/ET signaling pathway in an NPR1-dependent ISR manner [35,36,37]. Our demonstration that the HN-Q-8 strain could induce resistance to early blight resistance prompted us to investigate the expression of key genes of the JA/ET signal pathway and *NPR1* in potato plants treated with FLBS, BCS, or FL for 0–72 h.

**Figure 3:**
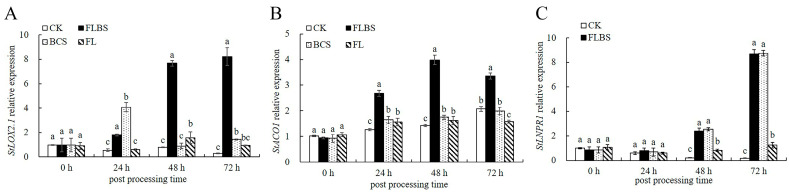
Effects of FLBS, BCS, or FL of the HN-Q-8 strain on the expression of *StLOX2.1* (**A**), *StACO1* (**B**), and *StNPR1* (**C**) genes related to ISR pathway in potato plants: (**A**,**B**). The results are presented as the means ± SDs (*n* = 3); columns labeled with different lowercase letters indicate statistically significant differences among treatments (*p* < 0.05).

The lipoxygenase (LOX) gene is one of the key genes in the JA signaling pathway [38]. Therefore, we first examined this gene’s expression in potato plants treated with FLBS, BCS, or FL. Compared with the control group, the FLBS-treated group displayed a significantly increased expression level of *StLOX2.1* at 24–72 h (Figure 3A). The expression level of *StLOX2.1* in the BCS group was significantly increased and peaked at 24 h. However, the expression level of *StLOX2.1* was decreased to control levels at 48 h, and then increased at 72 h. In contrast with the CK control group, no increased expression level of *StLOX2.1* in the FL group was observed at 24 h (Figure 3A), but this gene’s expression was activated at 48 h. Thus, FLBS had a strong effect on the induction of the expression of the key gene *LOX2.1* in the JA signaling pathway.

The expression of the 1-aminocyclopropane-1-carboxylate oxidase (ACO) gene is a major marker of ethylene biosynthesis and responsiveness [39]. Here, we also tested the expression levels of the *StACO1* genes of potato plants treated with FLBS, BCS, or FL of the HN-Q-8 strain. As shown in Figure 3B, the increased expression of the *StACO1* gene was only observed in the FLBS group from 24 to 72 h. Furthermore, the expression of the *StACO1* gene in the BCS and FL groups was induced at 24–48 h, but at 72 h, it was reduced to less than the control level. Therefore, we conclude that the addition of FLBS, BCS, or FL led to a degree of overexpression of the *StACO1* gene in the ET signaling pathway.

The nonexpressor of pathogenesis-related genes 1 (NPR1) genes are a class of resistance regulator genes, and their expression can reflect the resistance of plants [40]. Here, no increased expression level of the *StNPR1* gene in potato plants was observed for the FLBS, BCS, or FL groups at 24 h (Figure 2C). By comparison, the *StNPR1* gene in these three groups was activated at 48 and 72 h. In particular, at 72 h, significantly induced gene expression was noted in the FLBS and BCS treatment groups. Thus, FLBS, BCS, and FL treatments helped in inducing the expression of *StNPR1*.

### 3.4. Effect of Strain HN-Q-8 on the Growth of Potato Seedlings

**Figure 4:**
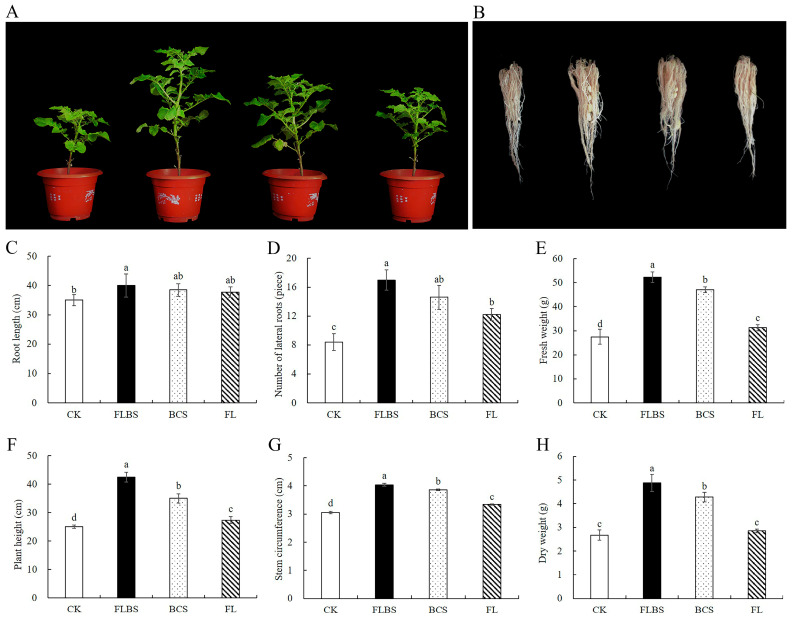
Effects of FLBS, BCS, or FL on the growth of potato seedlings. (**A**) Phenotypic changes in the above-ground parts. (**B**) Phenotypic change in below-ground parts. (**C**) Lateral root numbers. (**D**) Root length. (**E**) Fresh weight. (**F**) Plant height. (**G**) Stem diameter. (**H**) Dry weight. The results are presented as the means ± SDs (*n* = 5); columns labeled with different lowercase letters indicate statistically significant differences among treatments (*p* < 0.05).

As shown above, strain HN-Q-8 could induce resistance to potato early blight and also induce the expressions of the *StLOX2.1*, *StACO1*, and *StNPR1* genes. Next, we investigated whether the HN-Q-8 strain could promote the growth of potato plants by measuring the root length (RL), lateral root number (LR), stem diameter (SD), plant height (H), dry weight (DW), and fresh weight (FW). We found that the HN-Q-8 strain can promote the growth of potato plants. As shown in Figure 4A,F, FLBS had the strongest growth-promotion effect, and H was 1.7 times that of the control (Figure 4A,F); the root system was developed (Figure 4B), and the RL increased by 14.15% compared with the control (Figure 4C). The number of lateral roots increased by 104.38% (Figure 4D), and the SD, DW, and FW of the potato plants also increased significantly by 32.20%, 32.20%, and 90.22%, respectively. The effect of the HN-Q-8 BCS strain was inferior to that of FLBS, and it also had the effect of promoting plant growth, inducing root differentiation, and increasing dry-matter accumulation. After treatment with the HN-Q-8 BCS strain, the root length, number of lateral roots, stem diameter, plant height, dry weight, and fresh weight were increased by 9.87%, 73.81%, 25.33%, 33.20%, 23.55%, and 72.82%, respectively (Figure 4). The growth promotion effect of the FL treatment was weak, but compared with the control, it also significantly increased the number of lateral roots, plant height, stem diameter, and fresh weight of the potato seedlings (Figure 4). Thus, the HN-Q-8 strain has a growth-promoting effect, which could promote the growth of potato seedlings by interacting with potato roots.

### 3.5. Effect of Strain HN-Q-8 on Potato Tuber Yield

**Figure 5:**
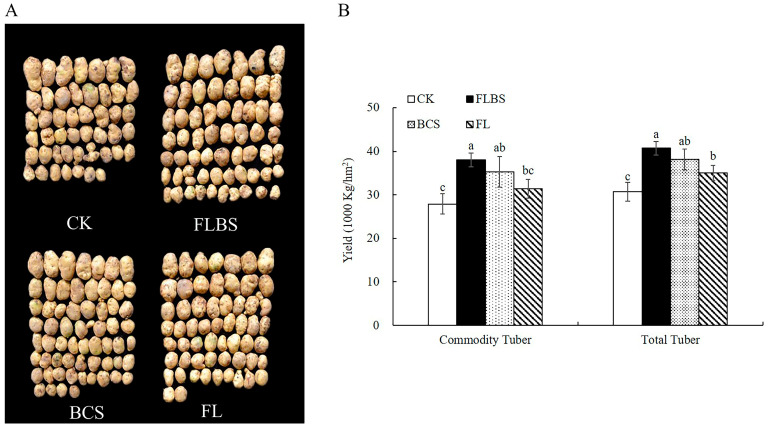
Effects of FLBS, BCS, or FL of strain HN-Q-8 on potato tuber yield. (**A**) Photographs of the effects of FLBS, BCS, or FL on potato tuber quality and yield. (**B**) Effects of FLBS, BCS, or FL on total potato tuber yield and commercial potato tuber yield. The results are presented as the means ± SDs (*n* = 3); columns labeled with different lowercase letters indicate statistically significant differences among treatments (*p* < 0.05).

Next, we tested whether this strain could increase potato tuber yield by performing field experiments. Compared with the controls, the FLBS, BCS, and FL groups displayed increased tuber yield (Figure 5A). Specifically, the maximum increased yield was observed for the FLBS group: a 36.38% and 35.83% increase for total yield and commercial potato yield (Figure 5B), respectively. Furthermore, the increased yield of the BCS group was 26.34% and 24.26% for the total and commercial weight tubers, respectively, which was significantly higher than that of the FL group. Thus, the HN-Q-8 strain can increase both potato tuber yield and commercial quality.

### 3.6. Effects of Strain HN-Q-8 on Plant Hormones in Potato

**Figure 6:**
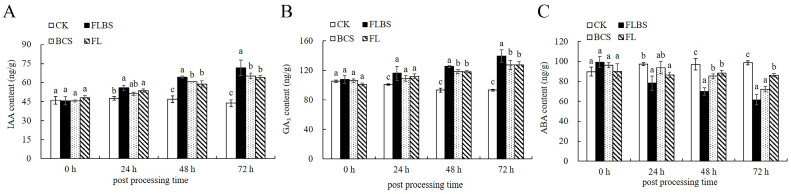
Effects of FLBS, BCS, or FL of strain HN-Q-8 on hormone content in potato plants. (**A**) IAA content; (**B**) GA_3_ content; (**C**) ABA content. The results are presented as the means ± SDs (*n* = 3); columns labeled with different lowercase letters indicate statistically significant differences among treatments (*p* < 0.05).

Plant hormones play an important role in regulating plant growth and development. In this experiment, changes in the levels of IAA, GA_3_, and ABA in potato leaves were measured at 0–72 h after treatments with FLBS, BCS, or FL. After the potato seedlings were treated, the contents of IAA and GA_3_ in the leaves increased significantly compared with the control, while the content of ABA decreased (Figure 6). Among them, the FLBS treatment had the most significant effect. At 72 h, the contents of IAA and GA_3_ in the leaves of this group increased by 55.69% and 49.15%, respectively, but ABA decreased by 37.53% (Figure 6). Thus, strain HN-Q-8 could regulate the growth of potato seedlings by promoting the synthesis of IAA and GA_3_ and reducing the synthesis of ABA. This accelerated the early development of potato plants and laid a foundation for the potato tuber yield increase.

### 3.7. Growth-Related Gene Expression Analysis

**Figure 7:**
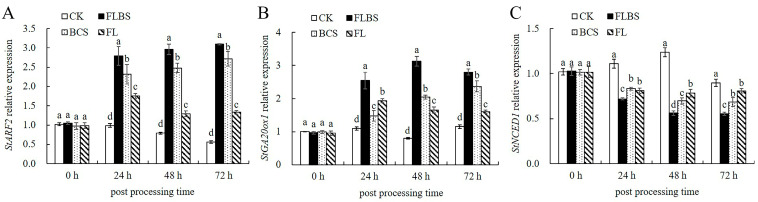
The effects of FLBS, BCS, or FL of strain HN-Q-8 on the expression of genes related to growth-related hormones in potato plants. (**A**) *StARF2*; (**B**) *StGA20ox1*; (**C**) *StNCED1*. The results are presented as the means ± SDs (*n* = 3); columns labeled with different lowercase letters indicate statistically significant differences among treatments (*p* < 0.05).

Because the plant hormone contents of potato leaves changed significantly, we measured the expressions of the IAA-, GA_3_-, and ABA-synthesis-related genes. Auxin response factors (ARFs) are a class of transcription factors that regulate IAA and auxin contents. The transcript level of *StARF2* was significantly increased within 24–72 h after treatment with FLBS, BCS, or FL. The FLBS and BCS groups showed a trend of gradual increase, which peaked at 72 h, and increased by 3.1- and 2.7-fold, respectively (Figure 7A), significantly inducing the synthesis of IAA. The expression of *StARF2* in the FL group peaked at 24 h by 1.7-fold, and then decreased (Figure 7A). The Gibberellin 20-oxidase (*GA20ox*) gene promotes the synthesis of active GA_3_. We found that, after treatment with FLBS, BCS, or FL, the expression of the *StGA20ox1* gene in the FLBS group peaked at 48 h, an increase of 3.13-fold. The expression level of the *StGA20ox1* gene in the BCS group continued to increase, peaking at 72 h by 2.37-fold. The FL group peaked at 24 h by 1.93-fold (Figure 7B), and then decreased slowly and stabilized. The 9-cis-epoxycarotenoid dioxygenase (NCED) is a key gene family for ABA synthesis in plants. After treatment with FLBS, BCS, or FL, the expression levels of the *StNCED1* gene in the FLBS, BCS, or FL groups were continuously inhibited, and decreased by 44.72%, 31.53%, and 21.76% at 72 h, respectively (Figure 7C). Thus, these treatments could induce the overexpression of *StARF2* and *StGA20ox1*, promote the synthesis of IAA and GA_3_, and inhibit the expression level of *StNCED1*, thereby inhibiting the synthesis of ABA. The results were broadly consistent with those for plant hormone levels.

### 3.8. Strain HN-Q-8 Increases the Chlorophyll Content and Root Activity of Potato

**Figure 8:**
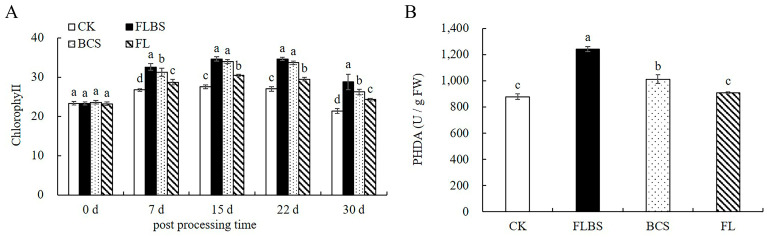
Effects of FLBS, BCS, or FL of the HN-Q-8 strain on chlorophyll content and root activity of potato. (**A**) Chlorophyll content of potato seedlings; (**B**) root activity of potato plants. The results are presented as the means ± SDs (*n* = 3); columns labeled with different lowercase letters indicate statistically significant differences among treatments (*p* < 0.05).

Because chlorophyll provides energy for plant metabolism, its content is related to photosynthesis. Therefore, we investigated whether the HN-Q-8 strain could enhance chlorophyll contents. As shown in Figure 8A, the chlorophyll content in the treatment group was higher than in the control group during the tested days (7–30 days). Among the treatment groups, the maximum potato chlorophyll content was found in the FLBS group, followed by the BCS and FL groups (Figure 8A). The HN-Q-8 strain could enhance potato plant chlorophyll contents.

Root activity is one of the important indexes to measure plant root function. Its activity directly affects plant growth and development and is of great significance for plant yield. Therefore, we examined the effect of the HN-Q-8 strain on the root activity of the potato plants. Compared with the control group, the FLBS and BCS groups had a 41.44% and 15.35% increase in root activity, respectively (Figure 8B). However, the FL group had the same root activity as the controls (Figure 8B). Thus, treatments with FLBS and BCS from the HN-Q-8 strain were able to enhance the root activity in these potato plants.

## 4. Discussion

Early blight is the one of the most important diseases threatening potato production, but chemical fungicides are still used to control it [1]. However, the long-term use of such agents will not only lead to bacterial resistance, but also pollute the environment [41]. The treatment of plants with *Bacillus* strains is capable of improving photosynthesis, inhibiting the growth of pathogens and inducing systemic resistance, which means it is an important biocontrol bacterial agent. For example, the *B. subtilis* strain ZD01 has a significant control effect on potato early blight [42], and *B. subtilis* CAS15 could cause systemic resistance to Fusarium wilt in pepper plants [43]. As part of the *Bacillus* genus, *B. velezensis* plays important roles in biocontrol [44]. For instance, *B. velezensis* also has a biological control function against diseases such as tobacco black shank and gray mold on pepper plants [45,46]. In our previous publication, we found that the *B. velezensis* HN-Q-8 strain has an obvious antagonistic effect on *A. solani*. The inhibition zone was 9.9 mm wide and, compared with the antibacterial effect of previous studies, the effect was extremely significant [42,45]. Here, we provided evidence that the *B. velezensis* HN-Q-8 strain can induce early blight resistance in potato plants, thus providing a potentially important biocontrol agent.

The activities of plant defensive enzymes are closely related to the disease resistance of plants [47]. SOD is an enzyme that can scavenge superoxide anion free radicals to protect the cell membrane structure [48]. POD is a protective enzyme in plants which can remove excessive reactive oxygen species and protect against damage [49]. CAT is ubiquitous in all tissues of plants, and its activity is related to metabolic intensity and disease resistance [50]. The activities of these three enzymes are positively correlated with plant disease resistance by effectively preventing the invasion and expansion of pathogens [34]. For example, Jiang et al. treated pepper plants with *B. velezensis* strains 5YN8 and DSN012; as a result, the activity of SOD, POD, and CAT increased by 13%, 27%, and 10%, respectively, and the strains imparted resistance to *Botrytis cinerea* [46]. Here, we found that the SOD, POD, and CAT activities of induced potato plants increased by 10–70% after treatment with FLBS, BCS, and FL, and the induction was faster and better than in the previous studies. The HN-Q-8 strain might enhance resistance to early blight by enhancing the activity of defense enzymes in potato plants.

*Bacillus* strains can activate the plant JA/ET signaling pathway by stimulating *NPR1* overexpression to produce systemic resistance to necrotrophic pathogens [35,36,37]. For instance, the *B. cereus* strain AR156 triggers the production of ISR through the JA/ET signaling pathway to enhance the resistance of *Arabidopsis thaliana* to *Pseudomonas syringae* [51]. Plants activate defense responses when injured to produce linolenic acid, and further generate JA through a series of enzymatic reactions including lipoxygenase (LOX) [38]. A key enzyme involved in the ethylene biosynthesis pathway is 1-aminocyclopropane-1-carboxylate oxidase (ACO), which directly catalyzes 1-aminocyclopropane-1-carboxylate to synthesize ethylene. Thus, the expression of the *ACO* gene is a signal of ethylene biosynthesis and response [39]. *NPR1* is a class of resistance-regulating genes, and their expression can reflect the resistance of plants [52]. Previous studies have shown that biocontrol bacteria can induce systemic resistance in plants by regulating the expression of disease-resistance-related genes in the JA/ET pathway. For example, Zhou et al. [28] treated tomato plants with the *Bacillus carinii* strain BH5, which activated the JA pathway and enhanced resistance to *Botrytis cinerea*. Ayaz et al. [53] used the *Bacillus atrophaeus* strain GBSC56 to induce tomato plant resistance to *Meloidogyne incognita* by activating the overexpression of *LOX* and *ACO1* genes. Wang et al. [54] treated sweet potato plants with the *Bacillus amyloliquefaciens* YTB1407 strain, which significantly increased the expression of *LOX* and *NPR1*, thereby enhancing the resistance to *Fusarium solani* and *Ceratocystis fimbriata*. Here, we showed that the expression of the *StLOX2.1*, *StACO1*, and *StNPR1* genes of potato plants could be induced by the *B. velezensis* strain HN-Q-8. This confirms that the JA/ET signaling pathway of potato plants could be rapidly activated faster and more strongly than in previous studies, thereby further enhancing the disease resistance of potato plants.

In addition to inducing plant disease resistance, *Bacillus* strains can promote plant growth and increase crop yield. For instance, the biomass of soybean plants was significantly increased after they were treated with the *Bacillus aryabhattai* strain SRB02 [55]. Furthermore, Ali et al. [56] treated potato plants with *Bacillus cereus*, which had significant effects on the yield compared with the control. In this work, after potato plants were treated with FLBS, the plant height, root length, fresh weight, dry weight, and other biomasses of the potatoes changed significantly, and the potato yields increased by 24–36%, much more than the 12% increase in Ali et al.’s study [56]. The results showed that the *B. velezensis* HN-Q-8 strain is an excellent growth-promoting strain, which could improve tuber yield by promoting the early accumulation of biomass.

Plant hormones play crucial roles in regulating plant growth. IAA can regulate plant cell division, cell elongation, and plant morphology. GA_3_ plays an important role in promoting stem elongation and leaf expansion. ABA accelerates leaf aging and promotes leaf abscission [55]. Moreover, auxin response factors (ARFs) are transcription factors that mediate responses to the plant hormone auxin [57]; gibberellin 20-oxidase (GA20ox) is a key rate-limiting enzyme in the biosynthetic pathway of GA [58]; 9-cis-epoxycarotenoid dioxygenase (NCED) is a key enzyme in ABA anabolism [59]. In this study, FLBS, BCS, or FL could promote the increase in contents of IAA and GA_3_ in potato plants and inhibit the synthesis of ABA, which could also regulate the expression levels of *StARF2*, *StGA20ox1*, and *StNCED1*. These change trends are consistent with that of the plant hormones, a result which is similar to that of Qin et al. [60]. Thus, the HN-Q-8 strain could promote the accumulation of biomass in an early growth stage and the formation of potato tubers in the late-growth stage by regulating the contents of plant hormones.

During the growth of plants, the chlorophyll content is related to photosynthesis, which provides energy for metabolism [61]. Plant root activity plays an essential role in the absorption of water and nutrients, which provide plants with energy from the soil, thereby promoting growth [62]. Ansari et al. [63] treated wheat seedlings with the *Bacillus licheniformis* B642 strain, which had significant effects on the plant chlorophyll content compared with the control. In addition, treatment with the *Bacillus tequilensis* JN-369 strain could significantly improve the content of chlorophyll and the root activity of rice plants [35]. In our study, the HN-Q-8 strain enhanced the potato plant chlorophyll content and stimulated root activity by improving the physiological metabolism of potato plants and increasing the absorption of soil nutrients.

Generally, *Bacillus* fermentation broth contains both living bacteria and secondary metabolites. Previous studies have shown that not only the living bacteria but their secondary metabolites can induce plant system resistance and promote growth [64]. For example, the antimicrobial lipopeptides fengycin and surfactin produced by some *Bacillus* strains can activate systemic resistance and promote growth in soybean plants [3]. In addition, Zhou et al. [28] found that fengycin H produced by the *Bacillus cabrialesii* strain BH5 could enhance the resistance of tomato plants to *Botrytis cinerea* to varying degrees. Here, we compared the roles of the FLBS, BCS, or FL containing secondary metabolites in inducing potato resistance to early blight and promoting growth. The FLBS treatment had the highest efficiency for inducing resistance to early blight and promoting growth, followed by BCS and FL, indicating that both strain HN-Q-8 living bacterial cells and secondary metabolites might associate with and affect the metabolism of potato plants, however, they should be used together because it is most the effective.

## 5. Conclusions

In conclusion, here we provide evidence that the *Bacillus* strain HN-Q-8 can induce potato plant resistance to early blight by regulating defensive enzyme activities and triggering the JA/ET pathway, as well as promote growth by regulating the contents of IAA, GA_3_, and ABA, enhancing the chlorophyll content, and stimulating root activity. Thus, our work has augmented the types of biocontrol agents available for potato cultivation. Based on the above results, further and deeper research on the molecular mechanism and regulatory network of the HN-Q-8 strain, which could induce early blight resistance and promote growth in potato, will be conducted in the future. Our study can provide vital information for the research and development of biocontrol bacteria.

## Figures and Tables

**Table 1:** Mass spectrometry parameters of three phytohormones.

Serial Number	Name	Parent Ion	Daughter Ion	Declustering Voltage	Collision Energy
1	IAA	173.9	130; 128	−49; −49	−12; −24
2	GA3	345	142.9; 238.9	−78; −90	−32; −20
3	ABA	264	153.1; 219.9	−60; −67	−16; −17

**Table 2:** Sequences of primer used for qPCR analysis.

Gene Name	Forward Primer	Reverse Primer
*StNPR1*	TATTGGCTGCACGAAGTCAGT	CGCACCAAATCCTTCAGCAAA
*StLOX2.1*	GCAGCTGTTAACTTTGGCCAA	CCACTCCCATTCTTCAGCTGT
*StACO1*	TGACAAAGTGAGTGGCCTTCA	CCTCAAGTTGGTCACCAAGGT
*StARF2*	GGGGATCTTCGTGTTGGAGTT	CCAAGCTGTTGCAAGTACACC
*StGA20ox1*	TGATGGTGTCACTGGCTATGG	TCGAGCATGTTCAATTGGGGA
*StNCED1*	GTACCGGAAAACCCAGTTTGC	CGAAAAGAGGGTTAGCTCCGT
*StActin7*	TTTGCTGGTGATGATGCTCCT	AGCTTCATCACCCACATAGGC
*StNPR1*	TATTGGCTGCACGAAGTCAGT	CGCACCAAATCCTTCAGCAAA

## Data Availability

The data presented in this study are available on request from the corresponding authors. The data are not publicly available due to privacy.
